# Insights into Orbital Symmetry: A Comprehensive Retrospective Study of 372 Computed Tomography Scans

**DOI:** 10.3390/jcm13041041

**Published:** 2024-02-12

**Authors:** Guido R. Sigron, Céline L. Britschgi, Brigitta Gahl, Florian M. Thieringer

**Affiliations:** 1 Department of Oral and Cranio-Maxillofacial Surgery and 3D Print Lab, University Hospital Basel, CH-4031 Basel, Switzerland; celine.britschgi@stud.unibas.ch (C.L.B.); florian.thieringer@usb.ch (F.M.T.); 2 Medical Additive Manufacturing Research Group (Swiss MAM), Department of Biomedical Engineering, University of Basel, CH-4123 Allschwil, Switzerland; 3 Surgical Outcome Research Center, Department of Clinical Research, University Hospital Basel, University of Basel, CH-4031 Basel, Switzerland; brigitta.gahl@usb.ch

**Keywords:** orbital volume measurement, automated segmentation, artificial intelligence, orbital symmetry, bony orbit, aging

## Abstract

**Background:** The operation planning and production of individualized implants with the help of AI-based software after orbital fractures have become increasingly important in recent years. This retrospective study aimed to investigate the healthy orbitae of 372 patients from CT images in the bone and soft tissue windows using the Disior™ Bonelogic™ CMF Orbital software. (version 2.1.28). **Methods:** We analyzed the variables orbital volume, length, and area as a function of age and gender and compared bone and soft tissue windows. **Results***:* For all variables, the intraclass correlation showed excellent agreement between the bone and soft tissue windows (*p* < 0.001). All variables showed higher values when calculated based on bone fenestration with, on average, 1 mL more volume, 0.35 mm more length, and 0.71 cm^2^ more area (*p* < 0.001). Across all age groups, men displayed higher values than women with, on average, 8.1 mL larger volume, a 4.78 mm longer orbit, and an 8.5 cm^2^ larger orbital area (*p* < 0.001). There was also a non-significant trend in all variables and both sexes toward growth with increasing age. **Conclusions:** These results mean that, due to the symmetry of the orbits in both the bone and soft tissue windows, the healthy orbit can be mirrored for surgical planning in the event of a fracture.

## 1. Introduction

The bony orbit is a complex anatomic structure with broad functional importance. The orbital shape and volume can be affected in patients with midfacial trauma, tumors, or congenital pathologies. Accurate reconstruction of its original shape, symmetry, and volume is essential for long-term functional and aesthetic prognosis [1,2,3,4]. Overestimation or underestimation of the volume may result in exophthalmos or enophthalmos [5]. Therefore, accurate three-dimensional orbital analysis and knowledge are essential for the diagnosis, treatment, and operation planning of orbital fractures [6]. Since the contralateral side is often used to compare reconstructions, it is desirable to know how symmetrical the human bony orbits are and how they change with age or gender [7]. Several studies have shown that the bony orbit and thus the volume changes with age [8,9]. However, there seems to be a gender difference where the remodeling processes occurs, which may affect the calculated volume depending on the measurement method [10].

Today’s orbital volume measurement methods typically require human assistance for the correct segmentation in each CT slice, which is a challenging procedure [11]. This manual or semi-automated segmentation is time-consuming and can be prone to operator error [12]. Automatic segmentation is up to 8.5 times faster per orbit than semi-automatic segmentation, as shown in the study by Jansen et al. (2016) [11]. Automatic segmentation takes an average of 38 s and semi-automatic segmentation up to 327 s when manual adjustments are made. Recently created and developed software solutions can automatically measure these orbital volumes using artificial intelligence and analyze their shapes from computed tomography. This fully automated method, based on deep learning, of measuring the orbit seems to be at least comparable to a human expert.

The study by Chepurnyi et al. (2020) compared different segmentation approaches. It was shown that the investigated segmentation methods have the same accuracy in the evaluation of volume differences between two orbits of the same patient, including defect areas and prolapsed soft tissue volumes, but not in the absolute values of the orbital volume due to the different determinations of the anterior closure [13]. Similar results were obtained by the research group of Haywood et al. (2021), who used a fully automated method to define the internal boundaries of the bony orbit in both CT and MRI images. Compared to a human expert, a high level of agreement was found in both images [14].

No study had compared symmetry in healthy orbits based on fully automated segmentation when this study was written. In all cases, segmentation was performed semi-automatically or manually [15,16]. Concerning changes with age, initial published studies used fully automated segmentation, but these were limited to the Asian population [17,18].

This study aimed to use the Bonelogic™ CMF Orbital software from Disior™ Ltd. (Helsinki, Finland) to measure healthy orbits in a fully automated manner to evaluate the symmetry of the right and left orbit and the gender-specific changes to the bony orbit with age, using the variables volume, length, and area. Another task of the research was to state the comparability of values between the bone window and the soft tissue window of computed tomography.

## 2. Materials and Methods

### 2.1. Patient Data

Initially, 712 CT images were selected, which were acquired between 01/2019 and 10/2020 at the University Hospital of Basel, Switzerland. All images were acquired with one of the following four CT scanners from Siemens Healthineers (Erlangen, Germany): SOMATOM Definition Flash, SOMATOM Definition Edge, SOMATOM Definition AS+, or SOMATOM Force. The slice collimation (cSL) was 0.6 mm for all devices. Two CT data sets in axial slices, one in the bone window and one in the soft tissue window, were exported as DICOM files for each scan. The bone window settings were 400 to 900 HU for WL and 2000 to 3200 HU for WW. The images in soft tissue window images had a WL of 40 HU and a WW between 120 and 300 HU. All exported CT images had a slice thickness of 0.75 mm.

Patients were retrospectively and randomly selected from all patients who underwent CT of the head during this period. Inclusion criteria were as follows: decision-making ability (>16 years), general consent for further use of data for research purposes, and complete imaging of the bony orbit. Orbits with pathology such as fracture, tumor, or previous orbital reconstruction were excluded. Patients were also excluded if the slice thickness was not 0.75 mm, and if more or fewer than two series were exported, one series in the bone window and one series in the soft tissue window. The above criteria reduced the number of CT images from an initial 712 to 372 from 186 patients. Of the patients included, 96 were male (52%) and 90 were female (48%). The mean age was 53 years, with a range from 16 to 88 years and with a standard deviation of 18 years.

The Ethics Committee EKNZ (Ethikkommission Nordwest- und Zentralschweiz, Basel, Switzerland) provided the required ethical approval with the corresponding code: 2022-00546.

### 2.2. Segmentation and Analysis

In this study, the three-dimensional (3D) medical imaging software “Bonelogic™ CMF Orbital software” (version 2.1.28) was used. It automatically creates a 3D model of the orbit based on CT or CBCT scans after the DICOM file is uploaded to the software. It was initially designed for patients with orbital fractures, where the software analyzes the shape, size, and location of the defective region. It also defines areas for reconstruction and can design patient-specific implants for surgery. In this study, the software was used to measure and compare healthy orbits. An advantage of the software is that it automatically determines the anterior orbital opening, making it user-independent.

**Figure 1:**
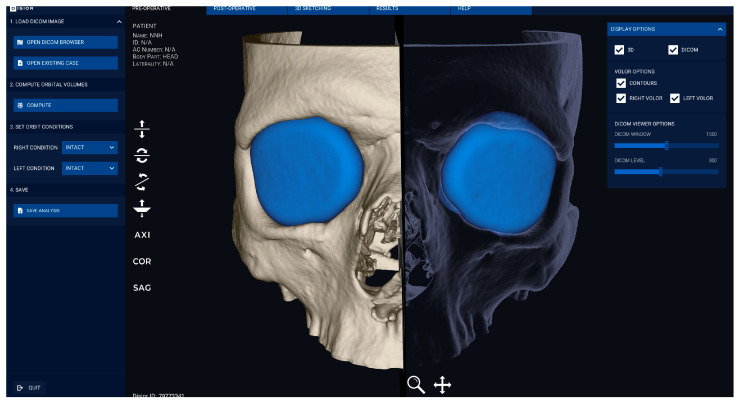
Automatic software segmentation of the orbits by the Bonelogic™ CMF Orbital software, by ball-shaped meshes that expanded and deformed until they made contact to the bony boundaries of the orbit.

The DICOM files were imported into the Disior™ Bonelogic™ CMF Orbital software (version 2.1.28) and reconstructed into a three-dimensional voxel map representing the bone situation. Voxel sizes varied from 0.28 × 0.28 mm to 0.52 × 0.52 mm. Based on the voxel data, ball-shaped meshes were inserted into both orbits. These meshes were iteratively expanded and deformed until they contacted the bony boundaries of the orbit, as shown in Figure 1.

For each imported CT image, the software calculates the parameters length (mm), surface area (anterior orbital opening, cm^2^), and volume (mL) for each of the right and left orbits. Consequently, there are two measurements per CT scan of a patient, each in both windows.

**Figure 2:**
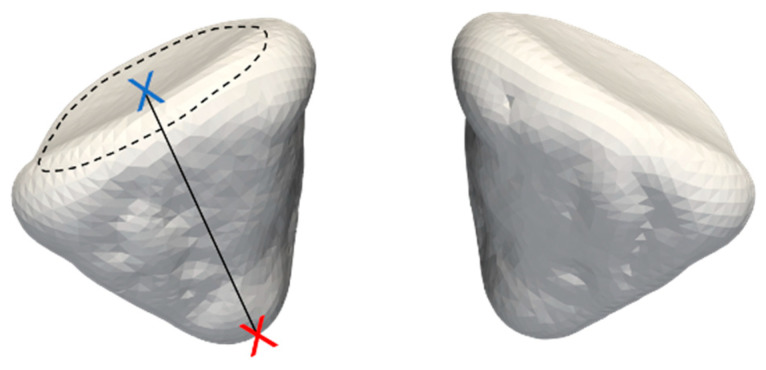
Presentation of the definition of the anterior closing (dashed line), the length (distance between the center point of the anterior closing and the most posterior point), and the volume.

Length is the distance from the center of the anterior closure to the farthest point of the orbital walls. The area is the sum of the areas of each triangle, comprising the surface mesh of the orbital walls, excluding the anterior closure. The volume is the sum of the volumes of each tetrahedron consisting of the volume mesh of the orbit. The parameters are visualized in Figure 2. 

Using this software, the anterior and posterior orbital closures are automatically defined and thus are independent of any person. The entire segmentation of the orbits is performed automatically without any user interaction.

### 2.3. Statistical Analysis

All analyses were calculated using Stata 16.0 (Stata Corp LLC, 4905 Lakeway Drive, College Station, TX, USA). To address whether orbital volume, length, and area are associated with age, the cumulated left and right orbital values were included as dependent variables into a linear mixed regression model, with patient age and gender as independent variables and patient as a random factor.

To assess the agreement of the reconstructions based on bones and the reconstructions based on soft tissue, the intraclass correlation coefficient (ICC) methods using a one-way random-effects model to estimate absolute agreement between individual measurements on the basis of reconstruction was applied. According to the notation of Shout and Fleiss, we used ICC (1.1). As suggested by Koo et al., ICC < 0.5 was interpreted as indicating poor agreement, between 0.5 and 0.75 was moderate, between 0.75 and 0.9 was good, and >0.9 was excellent agreement [19]. The agreement was visualized by using Bland–Altman plots. Continuous variables were summarized as mean and standard deviation (SD) after visually checking normal distribution, and categories as numbers with percentages. *p* values below 0.05 were considered statistically significant.

## 3. Results

### 3.1. Comparability of Soft Tissue and Bone Windows

The intraclass correlation coefficients showed excellent agreement between the two windows for all three parameters of the orbit, volume, area, and length (*p* < 0.001), as shown in Table 1. The average values in the bone window were 29 mL for the orbital volume, 40 mm for the length of the orbits, and 39 cm^2^ for the surface area. In the soft tissue window, the average values for the volume were 28 mL, for the length 39 mm (left) and 40 mm (right), and 38 cm^2^ for the surface area. Left and right orbits agreed equally for both bone and soft tissue windows and showed no statistically significant differences.

**Figure 3:**
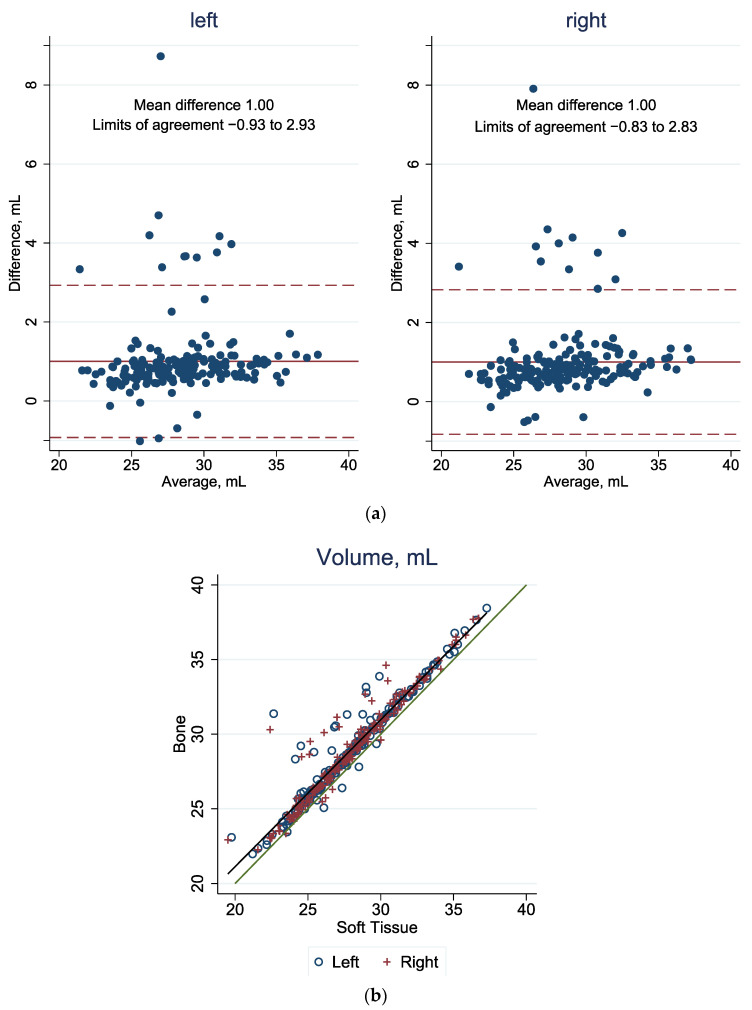
(**a**) Agreement of volume in bone and soft tissue windows: Bland–Altman plot, with the mean difference (solid red line) and the 95%limit of agreement (between the dashed red lines). (**b**) Agreement of orbital volume in bone and soft tissue windows. The green line represents the diagonal, and the gray line represents the best fit. See Appendix A Table A1 for corresponding numbers.

**Figure 4:**
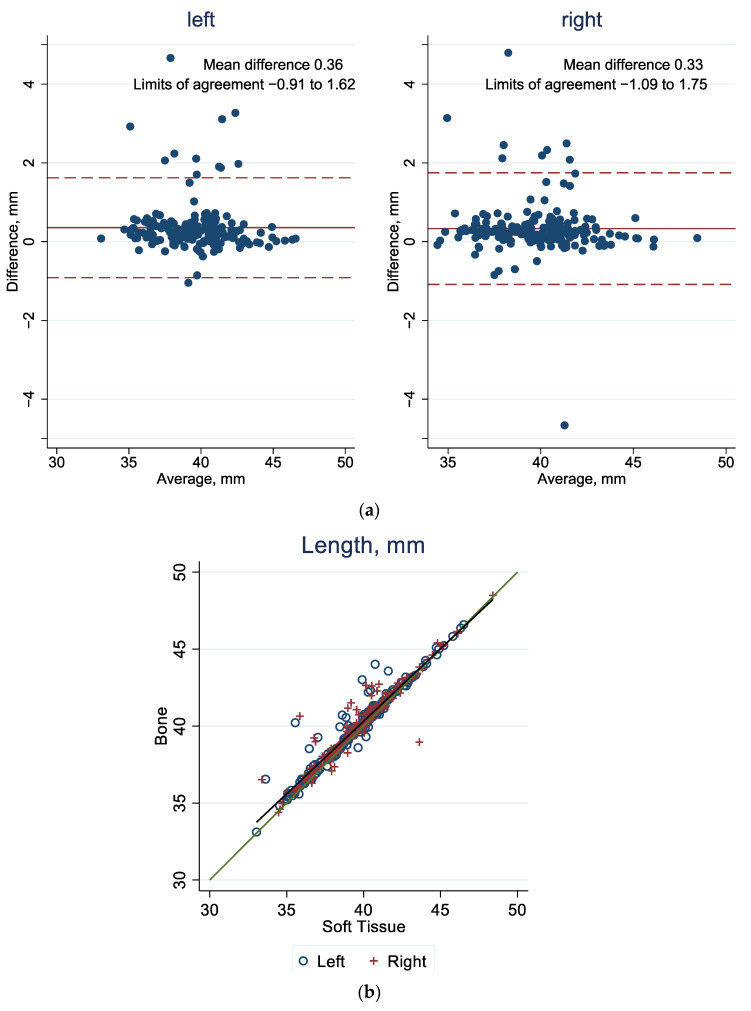
(**a**) Agreement of orbital length in bone and soft tissue windows: Bland–Altman plot, with the mean difference (solid red line) and the 95% limit of agreement (between the dashed red lines). (**b**) Agreement of orbital length in bone and soft tissue windows. The green line represents the diagonal, and the gray line represents the best fit. See Appendix A Table A1 for corresponding numbers.

**Figure 5:**
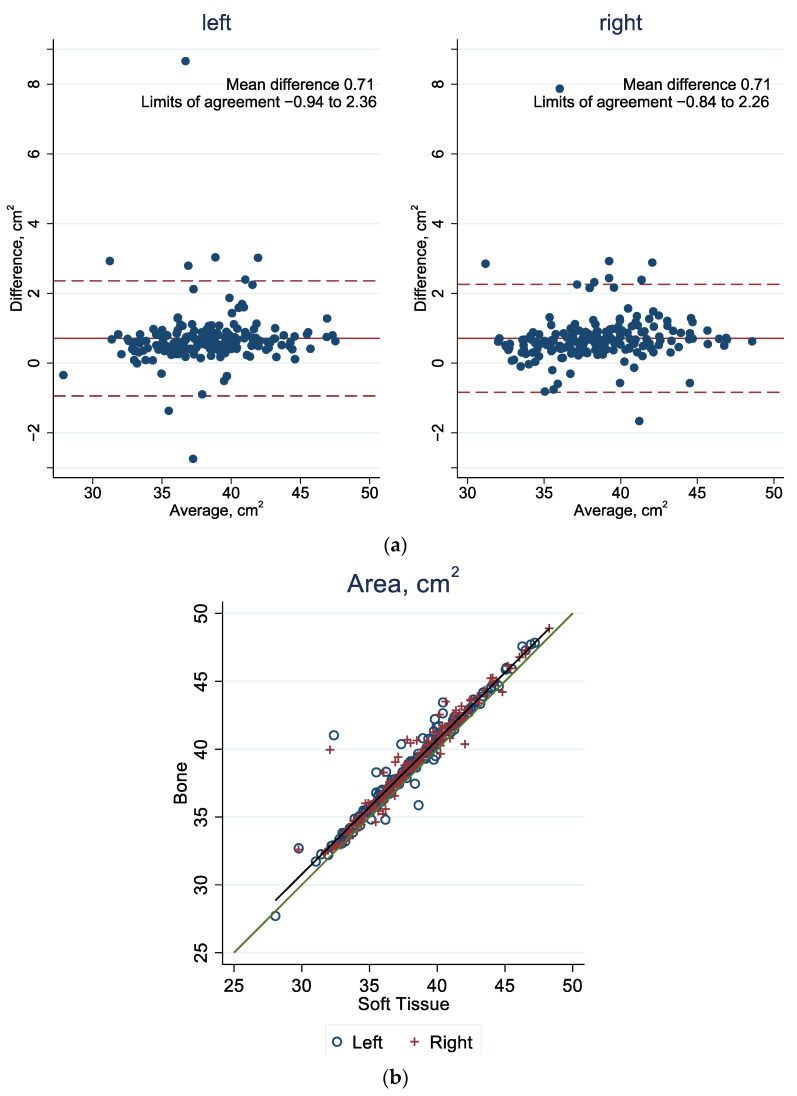
(**a**) Agreement of orbital area in bone and soft tissue windows: Bland–Altman plot, with the mean difference (solid red line) and the 95% limit of agreement (between the dashed red lines). (**b**) Agreement of orbital area in bone and soft tissue windows. The green line represents the diagonal, and the gray line represents the best fit. See Appendix A Table A1 for corresponding numbers.

In Figure 3a, Figure 4a and Figure 5a, in the Bland–Altman plots, the 95% limit of agreement is the area between the two dashed red lines. The blue dots represent the difference in value for each patient between the bone and the soft tissue windows. The red line represents the mean difference among all calculated values of the bone and soft tissue windows. In the scatter plots in Figure 3b, Figure 4b, and Figure 5b, all values are shown as one point representing the bone window value on the vertical axis and the value of the reconstruction based on the soft tissue window on the horizontal axis. Two sites are marked for each patient: a blue circle for the left orbit and a red cross for the right orbit. The green line forms the bisector, i.e., when both types of reconstruction correspond to the same value. Outliers can be observed in all graphical representations (Figure 3, Figure 4 and Figure 5). In these scenarios, the algorithm underestimates the measurements because there are pointed misrepresentations on the three-dimensional representation.

#### 3.1.1. Volume

Considering the volume of the orbit, the average value is 1 mL higher when the reconstruction is performed based on the bone window CT than when the reconstruction is based on the same CT image but in the tissue window, as shown in Figure 3a. The calculated mean difference is exactly 1 mL for the right and for the left orbits. Therefore, 6.99% of the values for the left orbit and 6.45% of the values for the right orbit are outside the 95% limits of agreement (left: −0.93, 2.93 and right: −0.83, 2.83). In Figure 3b, this difference can be clearly seen, as the points in the average are above the green line.

#### 3.1.2. Length

For orbital length, the mean difference from the bone window to the tissue window is 0.35 mm (left: 0.36 mm and right: 0.33 mm). The 95% limits of agreement are defined as −0.91, 1.62 for the left side and −1.09 and 1.75 for the right side. In all, 12 out of 186 values of all calculated lengths of the left orbits are outside these limits of agreements, which corresponds to 6.45%. For the lengths of the right orbits, 4.84% are outside the limits of agreement. The fact that the orbital lengths are on average longer when measured using the bone window CT is also supported in Figure 4b, where most points also lie slightly above the green line.

#### 3.1.3. Area

As can be seen in Figure 5a, the orbital surface area is on average 0.71 cm^2^ larger when the reconstruction is based on the CT image in the bone window as opposed to the soft tissue window. Here, 4.30% of the values are outside the 95% limits of agreement (left: −0.94, 2.36 and right: −0.84, 2.26), corresponding to 8 out of 186 CT scans. Figure 5b again shows the tendency for the area measured to be larger, based on the bone window.

#### 3.1.4. Symmetry

The left and right orbital measurements were in excellent agreement for volume, length, and area. The bone and soft tissue window measurements were comparable and showed no statistical difference (Table 2).

### 3.2. Effects of Age and Gender on Orbital Volume, Orbital Length, and Orbital Area

#### 3.2.1. Differences in Size of the Volume, Length, and Area of the Orbit by Gender

In our study cohort, male patients, regardless of age, had a mean 8.1 mL larger orbital volume in cumulated right and left orbital volume, a 4.78 mm longer orbit (cumulated right and left), and an 8.50 cm^2^ larger orbital area (cumulated right and left) than females. All these differences were highly statistically significant (Table 3).

#### 3.2.2. Development of the Volume, Length, and Area of the Orbit over Age According to Gender

**Figure 6:**
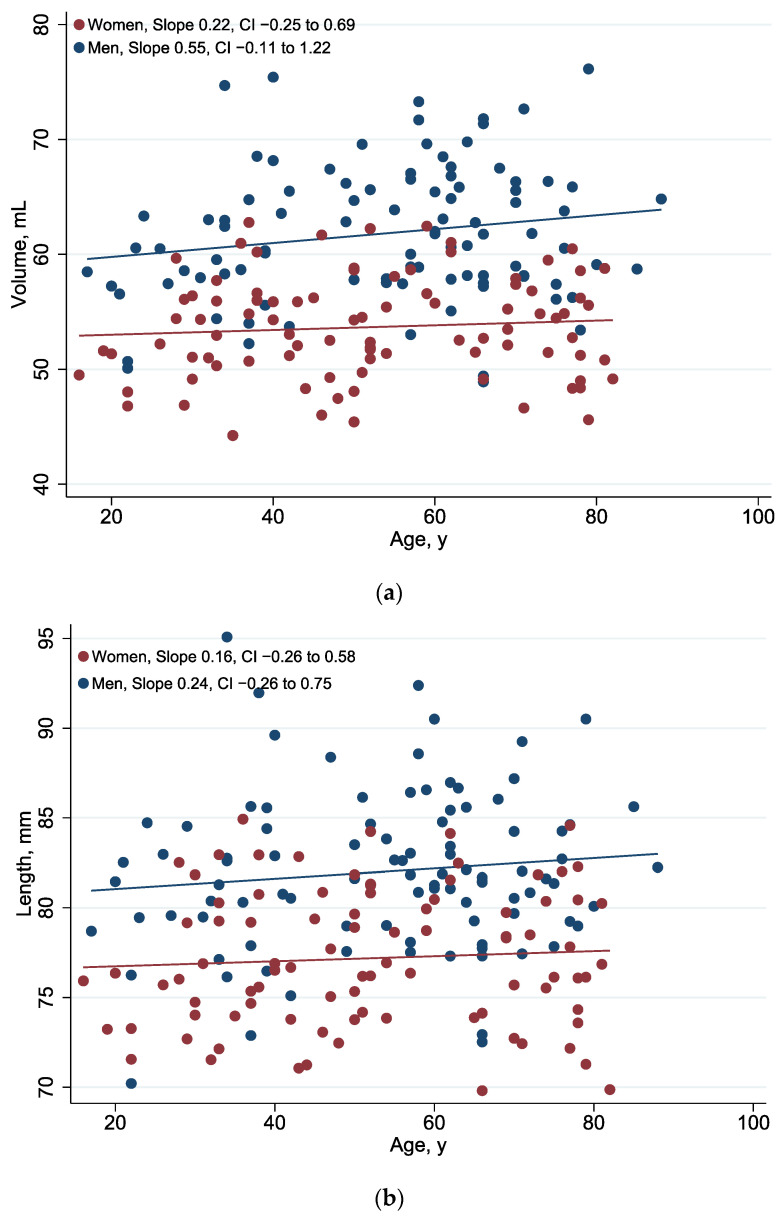

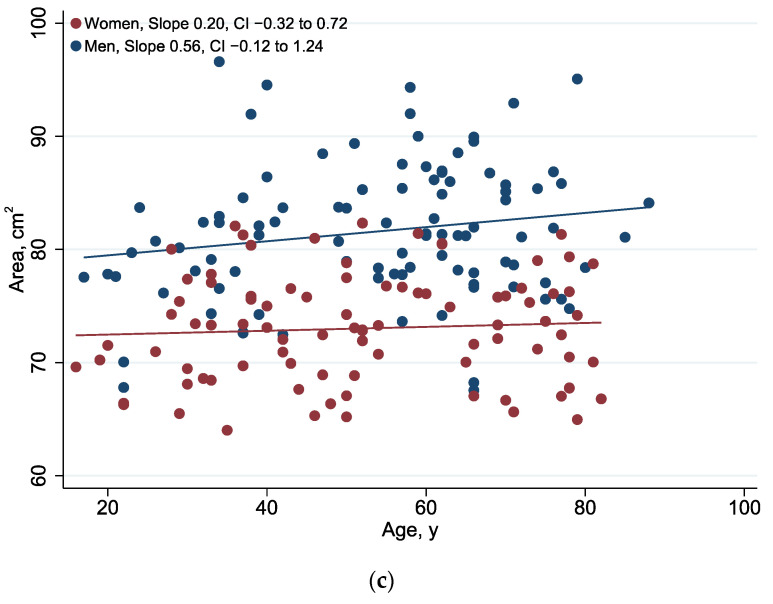
(**a**) Patient age and bone-derived orbital volume. See Appendix A Table A2 for corresponding numbers. (**b**) Patient age and bone-derived orbital length. See Appendix A Table A2 for corresponding numbers. (**c**) Patient age and bone-derived orbital area. See Appendix A Table A2 for corresponding numbers.

In both men and women, there is a tendency for the three variables of volume, length, and area of the orbit to increase with age (Figure 6, Table 4). The volume increases on average 0.21 mL per 10 years in women and 0.61 mL per 10 years in men (*p* = 0.357). The length of the orbit increases 0.14 mm per 10 years in women and 0.29 mm in men (*p* = 0.671). The area of the anterior orifice of the orbit increases on average 0.17 cm^2^ per 10 years in women and about 0.63 cm^2^ in men (*p* = 0.313).

We did not find an interaction of age and sex with respect to volume, length, and area, indicating that increase with age was parallel in female and male patients.

## 4. Discussion

The purpose of this study was to investigate how the symmetry and size of the bony orbit behave in males and females at different ages and to compare these values from the Bonelogic™ CMF Orbital software and CT images of the bony and soft tissue windows.

To date, the axial slices of the CT bone window have typically been used to create a 3D orbital model. However, sometimes only the axial soft tissue slices have been available. Organizing the orbital data can be time-consuming and cumbersome. Therefore, we wanted to determine if a 3D model created from soft tissue data would provide the same reconstruction quality as a model based on bone window data.

### 4.1. Comparability of Soft Tissue and Bone Window

To analyze the difference between the CT bone window and the CT soft tissue window, in our study, 186 pairs of orbits were compared using the Disior™ Bonelogic™ CMF Orbital software. For the three variables, orbital volume, orbital length, and orbital area, it was found that the software calculated higher values on average for reconstruction based on bone window CT images than reconstruction based on soft tissue CT images.

The volume in the bone window was on average 1 mL larger and the orbital length 0.35 mm longer than in the soft tissue window (*p* < 0.001).

To prevent the development of enophthalmos, the correct restoration of the orbital volume is particularly important. It is well-known in the literature that there is a direct correlation between the development of enophthalmos and an increase in orbital volume. On average, a volume increase of 1 mL results in an enophthalmos of 0.8–1 mm. Clinically, an enophthalmos of 1.6–2 mm and a volume difference of 2 mL are aesthetically and functionally relevant [10,19,20].

The 1 mL difference between the orbital volume when making a 3D model from the CT bone window or the CT soft tissue window is not clinically relevant.

It can be concluded that in everyday clinical practice, if only a CT soft tissue window image is available, it can save time and the need for a radiologist to organize and, if necessary, reconstruct the bone window image. With this background knowledge, a 3D model can be printed directly from the soft tissue window CT image, if required, and the orbital plate can be pre-bent before surgery or intraoperatively. This can make the process of pre-bending plates on a 3D model as described by Sigron et al. even more efficient [20].

A limitation of our study is that not only is the correct restoration of the orbital volume alone responsible for the development of an en- or exophthalmos, but also injuries and shrinkage of the periorbital tissue. This is not the subject of our study and is therefore not discussed.

### 4.2. Symmetry

Our study showed excellent agreement between the volume, length, and area of the right and left orbits in both the bone and soft tissue windows. There were no significant differences in any of the three variables. This confirms the strong positive correlation between right and left orbital volume shown by Walker et al. and in the study by Singh et al., where no significant differences between the two orbits were found in any parameter [15,21]. Other studies have shown a trend toward small differences in volume between the right and left orbits. Examples include the study by Lieger et al., with a mean difference of 600 ± 500 mL between the volumes (*p* = 0.039), and the study by Tandon et al., where a mean difference of 0.8 mL was calculated which, however, is not significant (*p* = 0.283) [16,22].

The average male orbital volume, right and left cumulated, is 8.1 mL larger than the female orbital volume. In the literature, the gender-specific differences vary and are also usually given per orbital side. Regensburg et al. indicated a comparable difference of 4 mL between women and men, and Amin et al. also calculated a difference of 3.08 mL [23,24]. A smaller difference between men and women was found by other authors, such as Andrade et al., with a difference of 1 mL [25].

Although there may be slight differences between the right and left bony orbits, we confirm the results of previous studies on the accuracy of using the opposite side as a reference when planning surgery for an orbital fracture [26,27,28].

### 4.3. Association of Patient Age and Gender with Orbital Volume, Length, and Area

In our study, there is a tendency for the bony orbit to change with age. The orbital volume, the area of the anterior opening, and the length of the orbit tend to increase. The increase seems to be greater in men than in women, but the results are not significant.

Similarities to our findings have been shown in other studies. Pessa et al. suggested a remodeling of the bony orbit in age as they discovered that with age the orbital rim moves backward in relation to the cornea [29]. Kahn et al. found an increase in volume with age, but without giving an exact increase value [10]. These results were also found by Li et al., who also used deep learning to make their measurements. Both Kahn and Li determined that the bone resorption of the superior orbital rim is more obvious than that of the inferior orbital rim in the aging process [17]. In the study by Chon et al., CT scans of the same individual were analyzed at a median time interval of 9.4 years. With an average 0.91 mL larger volume after 9.4 years, the scans displayed an even greater change in orbital volume with age than our results [8]. However, the measurements in their study were performed with a handheld device to measure the 2D orbital area. In future work, it would be enlightening to compare fully automated measurements on CT images of the same patients with a time difference.

In line with the study by Zhang et al., we saw a non-significant increase in both the length and the anterior opening of the orbit. Therefore, it can be assumed that the increase in orbital volume results from a combination of increases in both variables, length and area [30].

Orbital change with age is a complex process. Several reasons for a possible increase in orbital volume with age have been discussed. On the one hand, it has already been shown that the fat volume of the orbit increases significantly with age, which may be a possible cause of bony enlargement [31]. On the other hand, the change in the bony orbit may also be due to metabolic changes with age. As comprehensively described in the work of Cui et al., osteocytic changes in aging are now thought to lead to changes in the bone [32].

In this study, the focus is on the healthy bony orbit and thus the original condition. In practice, however, in addition to restoring the bony orbit as closely as possible to its original state, other relevant factors that influence the clinical outcome in the event of fracture must be considered. The timing of surgery after the fracture appears to have a significant impact on the outcome [33]. The meta-analysis by Damgaard et al. shows a significantly higher risk of persistent diplopia in patients who underwent surgery > 14 days after trauma [34]. A systematic review by Wevers et al. also identified fracture size, and localization as well as soft tissue involvement were also mentioned as the most relevant parameters for the clinical outcome [35]. In the study by Zimmerer et al., soft-tissue-related factors are assumed to hold great importance in predicting the clinical outcome. The authors suggest that in the future more attention should be paid to the destruction of infraorbital septa, extraocular muscles, and retroorbital fatty tissue, and that minimally invasive surgical techniques should be used to avoid this destruction [36].

There are limitations to this study that must be addressed. Patient characteristics, such as race or other pre-existing conditions, were not taken into account, which could influence the size of the orbit. These factors may also affect the size of the bony orbit and could be explored in a follow-up study. Another limitation of this study is that CT images from four different CT scanners were used, all of which are approved for clinical use. A real-life study with such a high number of cases using only one device would have been difficult to achieve as a single-center study. A comparison of the absolute values of orbital volume, length, and area among the CT devices for the same person would be of interest. However, it is ethically unjustifiable to conduct such a study with real patients due to the radiation exposure. The symmetry between the right and left orbits comes from the same image material and therefore the same CT device, and it is irrelevant for the statement on right/left symmetry.

In particular, the novelties of this study are the fully automated segmentation of the high number of images using modern AI-based tools and the subsequent calculation of orbital volumes, lengths, and areas. This study demonstrated high symmetry between the right and left sides regarding the volume, length, and area of all three parameters. This finding suggests that the healthy, mutual orbit can serve as a planning basis for orbital constructions.

Furthermore, the comparison of the volume, length, and area of the orbit between the soft tissue and bone windows is novel, where it could be shown that both windows can be used to create a 3D model.

## 5. Conclusions

In conclusion, our study demonstrates the practicality of mirroring the healthy contralateral side for orbital fracture surgery planning and 3D modeling, highlighting the symmetry of orbital structures. Importantly, we found no significant difference in model accuracy whether the bone or soft tissue window was used. This finding will facilitate clinical decision-making and may improve surgical outcomes in the treatment of orbital fractures.

## Figures and Tables

**Table 1:** Mean and intraclass correlation coefficients: reconstruction from bone vs. soft tissue window.

Variable	Bone, Mean (SD)	Soft Tissue, Mean (SD)	ICC (95% CI)	*p*
Volume left, mL	29 (3.3)	28 (3.2)	0.91 (0.51 to 0.97)	<0.001
Volume right, mL	29 (3.4)	28 (3.3)	0.92 (0.49 to 0.97)	<0.001
Length left, mm	40 (2.4)	39 (2.5)	0.96 (0.90 to 0.98)	<0.001
Length right, mm	40 (2.4)	40 (2.5)	0.95 (0.91 to 0.97)	<0.001
Area left, cm^2^	39 (3.5)	38 (3.4)	0.95 (0.79 to 0.98)	<0.001
Area right, cm^2^	39 (3.5)	38 (3.5)	0.96 (0.77 to 0.98)	<0.001

**Table 2:** Agreement of right and left orbits.

Modality	Variable	ICC (95% CI)	*p*
Bone window	Volume	0.98 (0.97 to 0.98)	<0.001
	Length	0.93 (0.91 to 0.95)	<0.001
	Area	0.95 (0.93 to 0.96)	<0.001
Soft tissue window	Volume	0.98 (0.97 to 0.98)	<0.001
	Length	0.93 (0.90 to 0.94)	<0.001
	Area	0.94 (0.92 to 0.96)	<0.001

**Table 3:** Mean (SD) and average difference of variables in men and women (cumulated left and right orbital values).

Size	Female (*N* = 90), Mean (SD)	Male (*N* = 96), Mean (SD)	Coefficient (95% CI)	*p*
Volume, mL	54 (4.4)	62 (5.9)	8.10 (6.59 to 9.61)	<0.001
Length, mm	77 (3.8)	82 (4.4)	4.78 (3.59 to 5.98)	<0.001
Area, cm^2^	73 (4.8)	82 (6.1)	8.50 (6.93 to 10.08)	<0.001

**Table 4:** Gender-specific increase (95% CI) in bony orbit per 10 years.

Variable	Women	Men	*p* *
Volume, mL	0.21 (−0.31 to 0.72)	0.61 (−0.09 to 1.30)	0.357
Length, mm	0.14 (−0.30 to 0.58)	0.29 (−0.23 to 0.81)	0.671
Area, cm^2^	0.17 (−0.38 to 0.72)	0.63 (−0.08 to 1.33)	0.313

* *p* was derived from interaction term age X sex.

## Data Availability

Data presented in this study are available on request from the corresponding author.

## Abbreviations

AI	Artificial intelligence
CBCT	Cone-beam computed tomography
CI	Confidence interval
CMF	Craniomaxillofacial
CT	Computed tomography
DICOM	Digital imaging and communications in medicine
HU	Hounsfield units
ICC	Intraclass correlation
WL	Window level
WW	Window width
3D	Three-dimensional

## Appendix A

The slope of volume and area did not show a deviation from one, indicating that bone-derived values were higher than soft-tissue-derived values by a constant amount, whereas the difference between bone- and soft-tissue-derived length values was larger in low than in high values.

**Table A1:** Slope and intercept of orbital volume, length, and area.

Variable	Slope (95% CI)	*p* *	Intercept (95% CI)	*p* *
Volume	0.98 (0.95 to 1.01)	0.307	1.44 (0.59 to 2.28)	0.001
Length	0.94 (0.91 to 0.97)	<0.001	2.66 (1.56 to 3.75)	<0.001
Area	0.99 (0.97 to 1.02)	0.469	1.05 (0.13 to 1.97)	0.026

* *p* value is based on H_0_: slope = 1 and H_0_: intercept = 0.

**Table A2:** Association of sex, age, and orbital assessments.

		Slope in Women	Slope in Men	Coefficient (95% CI)	*p*
Volume		0.22 (−0.25 to 0.69)	0.55 (−0.11 to 1.22)		
	Age per 10 y			0.22 (−0.35 to 0.79)	0.448
	Sex			6.22 (1.65 to 10.8)	0.008
	Age X Sex			0.33 (−0.49 to 1.15)	0.428
Length		0.16 (−0.26 to 0.58)	0.24 (−0.26 to 0.75)		
	Age per 10 y			0.16 (−0.30 to 0.62)	0.496
	Sex			4.45 (0.77 to 8.14)	0.018
	Age X Sex			0.08 (−0.58 to 0.74)	0.802
Area		0.20 (−0.32 to 0.72)	0.56 (−0.12 to 1.24)		
	Age per 10 y			0.02 (−0.40 to 0.80)	0.516
	Sex			6.54 (1.76 to 11.3)	0.007
	Age X Sex			0.36 (−0.49 to 1.22)	0.408

## References

[1] Wagner M.E.H., Gellrich N.-C., Friese K.-I., Becker M., Wolter F.-E., Lichtenstein J.T., Stoetzer M., Rana M., Essig H. (2016). Model-Based Segmentation in Orbital Volume Measurement with Cone Beam Computed Tomography and Evaluation against Current Concepts. Int. J. Comput. Assist. Radiol. Surg..

[2] Scolozzi P., Jaques B. (2008). Computer-Aided Volume Measurement of Posttraumatic Orbits Reconstructed with AO Titanium Mesh Plates: Accuracy and Reliability. Ophthal. Plast. Reconstr. Surg..

[3] Metzger M.C., Hohlweg-Majert B., Schön R., Teschner M., Gellrich N.-C., Schmelzeisen R., Gutwald R. (2007). Verification of Clinical Precision after Computer-Aided Reconstruction in Craniomaxillofacial Surgery. Oral Surg. Oral Med. Oral Pathol. Oral Radiol..

[4] Giovannetti F., Giona F., Ungari C., Fadda T., Barberi W., Poladas G., Iannetti G. (2009). Langerhans Cell Histiocytosis with Orbital Involvement: Our Experience. J. Oral Maxillofac. Surg..

[5] Hartwig S., Nissen M.-C., Voss J.O., Doll C., Adolphs N., Heiland M., Raguse J.D. (2019). Clinical Outcome after Orbital Floor Fracture Reduction with Special Regard to Patient’s Satisfaction. Chin. J. Traumatol. Zhonghua Chuang Shang Za Zhi.

[6] Sigron G.R., Barba M., Chammartin F., Msallem B., Berg B.-I., Thieringer F.M. (2021). Functional and Cosmetic Outcome after Reconstruction of Isolated, Unilateral Orbital Floor Fractures (Blow-Out Fractures) with and without the Support of 3D-Printed Orbital Anatomical Models. J. Clin. Med..

[7] Erdoğan K., Tatlisumak E., Ovali G.Y., Pabuşçu Y., Tarhan S. (2021). Age- and Sex-Related Morphometric Changes and Asymmetry in the Orbito-Zygomatic Region. J. Craniofac. Surg..

[8] Chon B., Zhang K.R., Hwang C.J., Perry J.D. (2020). Longitudinal Changes in Adult Bony Orbital Volume. Ophthal. Plast. Reconstr. Surg..

[9] Ugradar S., Lambros V. (2019). Orbital Volume Increases with Age: A Computed Tomography-Based Volumetric Study. Ann. Plast. Surg..

[10] Kahn D.M., Shaw R.B. (2008). Aging of the Bony Orbit: A Three-Dimensional Computed Tomographic Study. Aesthet. Surg. J..

[11] Jansen J., Schreurs R., Dubois L., Maal T.J.J., Gooris P.J.J., Becking A.G. (2016). Orbital Volume Analysis: Validation of a Semi-Automatic Software Segmentation Method. Int. J. Comput. Assist. Radiol. Surg..

[12] Diaconu S.C., Dreizin D., Uluer M., Mossop C., Grant M.P., Nam A.J. (2017). The Validity and Reliability of Computed Tomography Orbital Volume Measurements. J. Cranio-Maxillo-Fac. Surg. Off. Publ. Eur. Assoc. Cranio-Maxillo-Fac. Surg..

[13] Chepurnyi Y., Chernohorskyi D., Prykhodko D., Poutala A., Kopchak A. (2020). Reliability of Orbital Volume Measurements Based on Computed Tomography Segmentation: Validation of Different Algorithms in Orbital Trauma Patients. J. Cranio-Maxillo-Fac. Surg. Off. Publ. Eur. Assoc. Cranio-Maxillo-Fac. Surg..

[14] Hamwood J., Schmutz B., Collins M.J., Allenby M.C., Alonso-Caneiro D. (2021). A Deep Learning Method for Automatic Segmentation of the Bony Orbit in MRI and CT Images. Sci. Rep..

[15] Walker E.T., Lightfoot E., Walshaw E.G., Taylor R., Douglas J., Carter L.M., Parmar J.D. (2022). Quantitative Assessment of Bony Orbital Volume Symmetry: CT Analysis in the Uninjured Caucasian Population. Br. J. Oral Maxillofac. Surg..

[16] Lieger O., Schaub M., Taghizadeh E., Büchler P. (2019). How Symmetrical Are Bony Orbits in Humans?. J. Oral Maxillofac. Surg. Off. J. Am. Assoc. Oral Maxillofac. Surg..

[17] Li Z., Chen K., Yang J., Pan L., Wang Z., Yang P., Wu S., Li J. (2022). Deep Learning-Based CT Radiomics for Feature Representation and Analysis of Aging Characteristics of Asian Bony Orbit. J. Craniofac. Surg..

[18] Pan L., Chen K., Zheng Z., Zhao Y., Yang P., Li Z., Wu S. (2022). Aging of Chinese Bony Orbit: Automatic Calculation Based on UNet++ and Connected Component Analysis. Surg. Radiol. Anat. SRA.

[19] Koo T.K., Li M.Y. (2016). A Guideline of Selecting and Reporting Intraclass Correlation Coefficients for Reliability Research. J. Chiropr. Med..

[20] Sigron G.R., Rüedi N., Chammartin F., Meyer S., Msallem B., Kunz C., Thieringer F.M. (2020). Three-Dimensional Analysis of Isolated Orbital Floor Fractures Pre- and Post-Reconstruction with Standard Titanium Meshes and “Hybrid” Patient-Specific Implants. J. Clin. Med..

[21] Singh J., Rahman R.A., Rajion Z.A., Abdullah J., Mohamad I. (2017). Orbital Morphometry: A Computed Tomography Analysis. J. Craniofac. Surg..

[22] Tandon R., Aljadeff L., Ji S., Finn R.A. (2020). Anatomic Variability of the Human Orbit. J. Oral Maxillofac. Surg..

[23] Regensburg N.I., Wiersinga W.M., Van Velthoven M.E.J., Berendschot T.T.J.M., Zonneveld F.W., Baldeschi L., Saeed P., Mourits M.P. (2011). Age and Gender-Specific Reference Values of Orbital Fat and Muscle Volumes in Caucasians. Br. J. Ophthalmol..

[24] Amin D., Jeong J., Manhan A.J., Bouloux G.F., Abramowicz S. (2022). Do Racial Differences in Orbital Volume Influence the Reconstruction of Orbital Trauma. J. Oral Maxillofac. Surg..

[25] Andrades P., Cuevas P., Hernández R., Danilla S., Villalobos R. (2018). Characterization of the Orbital Volume in Normal Population. J. Cranio-Maxillofac. Surg..

[26] Pierrefeu A., Terzic A., Volz A., Courvoisier D., Scolozzi P. (2015). How Accurate Is the Treatment of Midfacial Fractures by a Specific Navigation System Integrating “Mirroring” Computational Planning? Beyond Mere Average Difference Analysis. J. Oral Maxillofac. Surg..

[27] Sozzi D., Gibelli D., Canzi G., Tagliaferri A., Monticelli L., Cappella A., Bozzetti A., Sforza C. (2018). Assessing the Precision of Posttraumatic Orbital Reconstruction through “Mirror” Orbital Superimposition: A Novel Approach for Testing the Anatomical Accuracy. J. Cranio-Maxillofac. Surg..

[28] Oh T.S., Jeong W.S., Chang T.J., Koh K.S., Choi J.-W. (2016). Customized Orbital Wall Reconstruction Using Three-Dimensionally Printed Rapid Prototype Model in Patients with Orbital Wall Fracture. J. Craniofac. Surg..

[29] Pessa J.E., Zadoo V.P., Yuan C., Ayedelotte J.D., Cuellar F.J., Cochran C.S., Mutimer K.L., Garza J.R. (1999). Concertina Effect and Facial Aging: Nonlinear Aspects of Youthfulness and Skeletal Remodeling, and Why, Perhaps, Infants Have Jowls. Plast. Reconstr. Surg..

[30] Zhang K.R., Chon B.H., Hwang C.J., Jellema L.M., Perry J.D. (2020). Comparison of Orbital Volume in Young Versus Senescent Human Skulls. Ophthal. Plast. Reconstr. Surg..

[31] Ugradar S., Manoukian N., Azhdam A., Le A., Chen J., Rootman D., Goldberg R.A., Lambros V. (2022). Orbital Aging: A Computed Tomography–Based Study of 240 Orbits. Plast. Reconstr. Surg..

[32] Cui J., Shibata Y., Zhu T., Zhou J., Zhang J. (2022). Osteocytes in Bone Aging: Advances, Challenges, and Future Perspectives. Ageing Res. Rev..

[33] Kasaee A., Mirmohammadsadeghi A., Kazemnezhad F., Eshraghi B., Akbari M.R. (2017). The Predictive Factors of Diplopia and Extraocular Movement Limitations in Isolated Pure Blow-out Fracture. J. Curr. Ophthalmol..

[34] Damgaard O.E., Larsen C.G., Felding U.A., Toft P.B., Von Buchwald C. (2016). Surgical Timing of the Orbital “Blowout” Fracture: A Systematic Review and Meta-analysis. Otolaryngol. Neck Surg..

[35] Wevers M., Strabbing E.M., Engin O., Gardeniers M., Koudstaal M.J. (2022). CT Parameters in Pure Orbital Wall Fractures and Their Relevance in the Choice of Treatment and Patient Outcome: A Systematic Review. Int. J. Oral Maxillofac. Surg..

[36] Zimmerer R.M., Gellrich N.-C., Von Bülow S., Strong E.B., Ellis E., Wagner M.E.H., Sanchez Aniceto G., Schramm A., Grant M.P., Thiam Chye L. (2018). Is There More to the Clinical Outcome in Posttraumatic Reconstruction of the Inferior and Medial Orbital Walls than Accuracy of Implant Placement and Implant Surface Contouring? A Prospective Multicenter Study to Identify Predictors of Clinical Outcome. J. Cranio-Maxillofac. Surg..

